# Retraction: Relationship between perceived occupational stress and psychological well-being among secondary school heads in Khyber Pakhtunkhwa, Pakistan

**DOI:** 10.1371/journal.pone.0322666

**Published:** 2025-04-14

**Authors:** The PLOS One Editors

Following the publication of this article [1], concerns were raised about similarity in study design and text overlap with multiple articles from the same author group [2–6], and about compliance with PLOS policy on Human Subjects Research.

Documentation provided by the corresponding author during post-publication discussions were not sufficient to demonstrate that the study was prospectively approved by a research ethics committee or institutional review board (IRB). The corresponding author stated that there is no formal IRB or equivalent at their institution.

The corresponding author confirmed during post-publication discussion that the articles [1–6] reported data from the same project but focused on different variables and outcomes, clarifying that the same sample population was used in all articles, and the same occupational stress results were reported in this article [1] and [3,4,6]. The data collection start date is not uniform across all articles [1–6], and the corresponding author provided conflicting information in post-publication discussions regarding the correct start date. The reuse of data was not declared in the published articles. In May 2022, they added a comment [7] to this article [1] to acknowledge the related studies and include citations to the articles that were published earlier [2,3].

There is extensive text overlap between this article [1] and a previous publication by the same group [3] which was not cited. In addition, Fig 1 and Tables 1 and 2 are similar in [1,3,4]. The corresponding author stated that the similarity is due to the reuse of data in these articles.

The *PLOS One* Editors retract this article due to concerns about compliance with PLOS policies on Human Subject Research and Plagiarism.

QS did not agree with the retraction. IH, SS, MAS, and SAR either did not respond directly or could not be reached.

## References

[1] Suleman Q, Hussain I, Shehzad S, Syed MA, Raja SA. Relationship between perceived occupational stress and psychological well-being among secondary school heads in Khyber Pakhtunkhwa, Pakistan. PLoS One. 2018;13(12): e0208143. doi: 10.1371/journal.pone.0208143 30540807 PMC6291082

[2] Suleman Q, Hussain I. Job satisfaction among secondary-school-heads: a gender based-comparative study. Education Sciences. 2018;8(1):28. doi: 10.3390/educsci8010028

[3] Suleman Q, Hussain I, Shehzad S. Relation of occupational stress and job satisfaction: a study of secondary school heads in Khyber Pakhtunkhwa, Pakistan. Global Social Sciences Review. 2018;III(II):237–72.

[4] Ishtiaq H, Suleman Q, Jumani NB. Occupational stress among secondary school heads: a gender based comparative study. Journal of Education and Educational Development. 2020;5(2):138. Available from: http://jmsnew.iobmresearch.com/index.php/joeed/article/view/138

[5] Suleman Q, Syed MA, Mahmood Z, Hussain I. Correlating emotional intelligence with job satisfaction: evidence from a cross-sectional study among secondary school heads in Khyber Pakhtunkhwa, Pakistan. Front Psychol. 2020;11:240. doi: 10.3389/fpsyg.2020.00240 32231604 PMC7083110

[6] Suleman Q, Syed MA, Khattak AZ, Kayani KM, Zaman Z, Khan I. The relationship between emotional intelligence and occupational stress among secondary school heads in Khyber Pakhtunkhwa, Pakistan. International Journal of Innovation, Creativity and Change. 2021;15(7):953–72.

[7] Suleman Q. Comment “ Updating Article Content with Citations of Previously Published Work.” 2022 [cited 2024 Jan 3]. Available from: https://journals.plos.org/plosone/article/comment?id=10.1371/annotation/102db5a3-a43b-495d-ab78-47d5fd7845f4

